# Pilot Testing of an Area-Wide Biological Control Strategy against the Coffee Berry Borer in Colombia Using African Parasitoids

**DOI:** 10.3390/insects14110865

**Published:** 2023-11-09

**Authors:** Pablo Benavides, Zulma Nancy Gil, Luis Eduardo Escobar, Lucio Navarro-Escalante, Peter Follett, Hilda Diaz-Soltero

**Affiliations:** 1 Department of Entomology, National Coffee Research Center, Cenicafé, Manizales 170009, Colombia; zulma.gil@cafedecolombia.com (Z.N.G.); luis.escobar@cafedecolombia.com (L.E.E.); lucionavarroe@gmail.com (L.N.-E.); 2 USDA-ARS, Daniel K. Inouye U.S. Pacific Basin Agricultural Research Center, 64 Nowelo St., Hilo, HI 96720, USA; peter.follett@usda.gov; 3 Animal and Plant Health Inspection Service, United States Department of Agriculture, District of Columbia (USDA), Washington, DC 20250, USA; hilda.diaz-soltero@usda.gov

**Keywords:** biological control, Colombian coffee, IPM, *Hypothenemus hampei*, *Phymastichus coffea*, *Prorops nasuta*

## Abstract

**Simple Summary:**

The coffee berry borer (CBB), so called “broca del café”, is originally from Africa and invaded Colombia in 1988. Classical biological control was attempted through the importation of African parasitoids, but after repeated releases, most of the parasitoid species did not become established. The objective of this research was to determine if releases of the parasitoid *Prorops nasuta* in CBB dispersal coffee plots, followed by releases of the parasitoid *Phymastichus coffea* in CBB colonization coffee plots within coffee farms, could lead to an overall decrease in the damage caused by this pest to coffee berries. The results showed that CBB populations decreased from the dispersal, and in the colonization coffee plots, resulted in lower levels of CBB infestation in coffee berries in the field and reduced crop loss. Well-timed and targeted release of these African parasitoids could be used in an area-wide control program against the CBB to lower population levels, reduce crop damage, and replace the use of chemical insecticides in an integrated pest management (IPM) program.

**Abstract:**

The coffee berry borer (CBB), *Hypothenemus hampei* (Ferrari, 1867) (Coleoptera: Curculionidae: Scolytinae), native to Africa, is a major global insect pest of coffee. It has invaded many coffee production areas around the world that do not have natural enemies. In this study, two African parasitoids, *Prorops nasuta* Waterston (Hymenoptera: Bethylidae) and *Phymastichus coffea* Waterston (Hymenoptera: Eulophidae), were mass-reared for field release against *H. hampei* in Chinchiná, Colombia. More than 1.5 million wasps of each species were released on a 61-hectare coffee farm in replicated plots, resulting in parasitism rates of up to 7.7% for *P. nasuta* and 56.3% for *P. coffea*. This led to a maximum reduction in *H. hampei* field populations of 81% from dispersal coffee plots (old coffee crops before plant stumping) and 64.3% in colonization coffee plots (new coffee crops with active growing and fruiting plants) within the farm. As a result of this area-wide strategy, the percentage of CBB-infested coffee berries in colonization coffee plots decreased from 51.1 to 77.5% compared to coffee plots without parasitoid releases. This approach offers a promising alternative to the use of chemical insecticides and could be integrated into current pest management programs to control *H. hampei*.

## 1. Introduction

Coffee represents one of the most important crops for the Colombian economy, with a planted area of 877,144 hectares, a total production of 13.89 million bags of 60 kg, and a contribution to the agricultural gross domestic product of 13.6% [1]. Currently, the crop is distributed in 602 municipalities, with 542,000 coffee-growing families that depend economically on coffee, and generates close to 2 million indirect jobs. In total, 84% of Colombian coffee farming has been planted with varieties resistant to coffee leaf rust such as Cenicafé1 and Castillo^®^; the average age of the coffee trees in the country is 6.89 years and the planting density is 5296 plants per hectare [2]. Unlike most coffee-producing countries, there are two harvests every year in Colombia, which vary in proportion depending on the latitudes.

Worldwide, coffee production is limited by the damage caused by the coffee berry borer (CBB), *Hypothenemus hampei* (Ferrari) (Coleoptera: Curculionidae: Scolytinae), which affects the physical and sensory quality of the coffee grain [3,4]. This insect is native to Africa and was introduced to South America in 1913 in Brazil [5], even though it was not until 1924 that damage was recorded on a farm in São Paulo [6]. Its spread was facilitated by the tropical climate, and it subsequently reached several other coffee-producing countries. It was reported in Colombia in 1988 in the southern part of the country in the Department of Nariño [7].

In Colombia, the climatic conditions of the coffee-growing area ensure the permanent availability of coffee fruits for the development of the CBB [8]. The damage that the insect has caused to Colombian coffee farming since its arrival in the country in 1988 is related to the direct affection to the grain, yield reduction, and quality and income lowering for coffee growers [7,9]. Although, in Colombia, the insect remains at tolerable levels, due to the implementation of an integrated management strategy with emphasis on cultural control [9,10], the problem persists, control costs increase, and pressure from the borer sometimes makes the coffee activity an economic challenge.

Regarding the costs to control the CBB in Colombia, Duque and Baker [11] estimate that these would amount to 16 million dollars annually if the CBB is controlled at levels of 3% infestation. If national production had a 10% damage, the losses would be around USD 66 million; but if the borer were not controlled, the damage could be estimated at 25% of the cherries damaged with losses of up to USD 180 million. If it is assumed that the cost of controlling the borer and keeping it at 5% is USD 100 per hectare, then the cost of controlling it on 500,000 hectares would be USD 50 million. If the losses caused by that 5% of infestation levels are added to this, the losses would be USD 75 million per year.

The spread of CBBs in Colombia is promoted by the periodic renovation of coffee plantations, which involves stumping of mature plants (or their removal) or planting new coffee plants. This process occurs about every 5 years, so the stumped or new plants start flowering again 12 to 16 months later. It has been estimated that 2.6 to 3.6 million adult CBBs per hectare disperse after removal of their host plants during renovation [12]. These adults may fly for several kilometers if wind conditions are favorable [12].

In order to minimize the dispersal of those millions of CBBs from stumped fields, Cenicafé recommends to leave trees, for around two to three months after, as traps in the borders and the middle of those coffee crops [10].

The adult females CBBs bore into the coffee berry, to lay their eggs, and offspring develop later. The CBB passes most of its lifetime inside the coffee berry, except for a short period of time when adult females disperse by flight in seek of new berries for food and reproduction [13]. The cryptic habits of this insect make it difficult to control. In Colombia, an integrated management strategy was developed that is based on the implementation of cultural, chemical, and biological control strategies according to the phenology of the crop and the behavior of the insect in the field [10,14,15]. Other natural enemies into a biological control approach shows potential as an environmentally friendly alternative [16].

Various natural enemies of the CBB have been reported and evaluated as an alternative for its control, including parasitoids, predators, entomopathogenic fungi, and nematodes [17]. *Beauveria bassiana* fungi could be used when the CBB adults are colonizing green fruits in the field following the recommendations used for chemical control [18], due to its action on the CBB individuals that are flying from the fruits onto the ground and those that are attacking the fruits [19]. Predators manage to enter through the perforations made by the CBBs and reduce the population inside the coffee fruits [20].

Seven parasitoids related to the CBB are described: four from its native range in Africa, and three from the Americas. Three of the African species—*Prorops nasuta* Waterston, *Cephalonomia stephanoderis* Betrem, and *Phymastichus coffea* LaSalle—have been introduced to Colombia [21]. The first two are ectoparasitoids in the Bethylidae family. Both attack all life stages of the CBB within infested coffee berries, acting both as predators and parasitoids. As a predator, the wasps suck the hemolymph from the adult CBBs, and consume eggs first, and the larvae second. Eggs are laid on prepupae and pupae, where they develop as parasitoids. *Prorops nasuta* and *Cephalonomia stephanoderis* were introduced to Colombia between 1989 and 1990 from colonies maintained in Ecuador that originated in Kenya and Togo, respectively [21]. Bacca [22] showed that *P. nasuta* quickly reduced coffee borer populations, killing all stages of the host via predatory and parasitic behavior.

In Colombia, around 560 million individuals of *P. nasuta* and 2 billion *C. stephanoderis* individuals were released expecting establishment in the field as permanent natural controllers. Maldonado et al. [23] collected samples of coffee infested by CBBs in 80 farms in 17 municipalities in the departments of Nariño, Quindío, Risaralda, Caldas and Norte de Santander, after six years of the last releases and 15 years after starting the program, in order to corroborate the establishment of these wasp species. *C. stephanoderis* was not found in the samples evaluated, while *P. nasuta* was recorded in all departments in 65% of the farms, in an altitudinal range between 1150 and 1840 m. The results obtained in the southern department of Nariño stand out, where this parasitoid was found in all the farms with parasitism of up to 50%. It is demonstrated that *P. nasuta* was superior in its capacity to adapt to the conditions of the Colombian coffee ecosystem, becoming established and contributing to the natural control of borer populations in the field.

The third species introduced in Colombia, *P. coffea* (Hymenoptera: Eulophidae), is a primary, gregarious, idiobiont endoparasitoid of CBB adults [24]. Its behavior is different from that of *P. nasuta* and *C. stephanoderis* as it directly attacks adults while they are colonizing coffee berries, preventing parasitized adults from boring into the berries to reproduce [23]. This parasitoid wasp was discovered in 1988 in Togo, West Africa, and was introduced to Colombia in 1996, after being examined in quarantine in England. These three wasp species are relatively host-specific, making them suitable for use in integrated pest management (IPM) of CBBs. Methods for mass rearing, releasing, and evaluating the impact as biological control agents of these three species have been assessed in Colombia [3,21,23,25].

An area-wide approach to CBB management may be possible using targeted and well-timed releases of parasitoids. The objective of this study was to conduct area-wide multiple release pilot trials of *Prorops nasuta* and *Phymastichus coffea* for the control of the CBB in Colombia. Thus, the releases of *P. nasuta* would lower CBB populations from dispersal patches (stumped coffee crops) and the timing releases of *P. coffea* would reduce the rate of CBB colonization on nearby coffee crops. The first due to its high predatory behavior and the second for its parasitic capacity in adults attacking fruits. Thus, this approach would lead to an overall decrease on the CBB infestation percentage in the field at an area-wide level.

## 2. Material and Methods

### 2.1. Study Area

#### 2.1.1. Overview of Sites and Management

**Figure 1:**
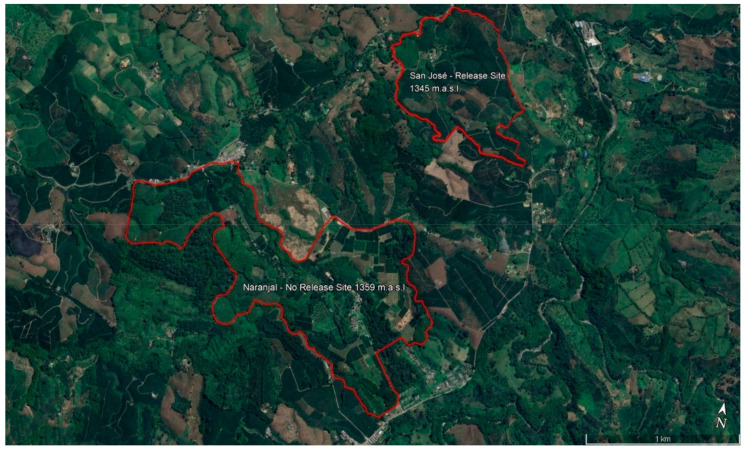
Location of the coffee farms used as study sites. The San Jose and Naranjal farms are located in the same geographical basin and at the same altitude.

A pilot test of the area-wide control of CBBs was carried out in 2021 and 2022 on two coffee farms in the Department of Caldas, Colombia: (i) the parasitoid release site was the San Jose farm, located in the municipality of Chinchiná, at 1350 m above sea level, with an average annual temperature of 21.6 °C, average relative humidity, 80.6%; annual precipitation, 2990 mm, 1537 h of sunshine/year, 4°59′22.89″ N; 75°38′53.70″ W (coordinates), and an area of 61 hectares of sun exposed coffee; and (ii) the control non-release site was the Naranjal Experimental Station, located in the municipality of Chinchiná, at 1381 m above sea level, with an average annual temperature of 21.6 °C, average relative humidity, 80.6%; annual precipitation, 2990 mm, 1537 h of sunshine/year, 4°58′02″ N; 75°39′56″ W (coordinates), and an area of 94.7 hectares of sun exposed coffee (Figure 1).

Both farms present two annual harvests: main harvest (70%) flowering between January and March; mid harvest (20%) flowering between August and September; and the 10% rest occurs inter harvesting. The coffee crops were planted with Cenicafe1 and Castillo^®^ varieties (National Coffee Research Center Cenicafé, Manizales, Colombia) resistant to coffee leaf rust, with a planting density between 6500 and 7700 plants per hectare.

Integrated weed management was carried out on both farms, which included control with herbicides in patches with highly interfering weed species; moreover, fertilization was based on soil analysis in each farm and coffee lot, and finally, integrated management of the CBB was carried out, including cultural control practices as the basis for that IPM program and chemical control for coffee lots on third, fourth, and fifth harvesting year. For those coffee lots in the first two harvesting years, no control, rather than timely harvesting and sanitization after harvest seasons, was performed. In the dispersal patches of both farms, the sanitary harvesting was carried out before stumping the plants, as well as leaving trap trees as recommended. *B. bassiana* was not sprayed at this moment as recommended since it is not a well adopted strategy and for the negative effect on the parasitoids.

Both farms use the coffee production system recommended in Colombia to maintain high levels of productivity, which begins with the establishment of production cycles of a maximum of five harvest years. For this, rotation of 20% of the coffee plots are renovated annually. Thus, the older coffee plantations become a reservoir of CBBs, from which high beetle numbers disperse to colonize coffee berries in the nearby coffee crops within the farm.

#### 2.1.2. Designation of Release, Control No Release Locations, and Source of Parasitoids

The San Jose farm received releases of two species of CBB parasitoids, *P. nasuta* and *P. coffea*. Both species were reared on washed *Arabica* coffee seeds (known as parchment coffee) as per Portilla et al. [26], Bustillo et al. [25], and Orozco [27]. Parasitoids were reared at the Biocafe Biological Supplies laboratory, located in La Quiebra del Naranjal village, Chinchiná, Caldas, Colombia [28]. Coffee crops at the Naranjal Experimental Station served as the reference control, where no parasitoids releases were performed.

#### 2.1.3. Definitions Dispersal and Colonization Coffee Plots in Study Areas

**Figure 2:**
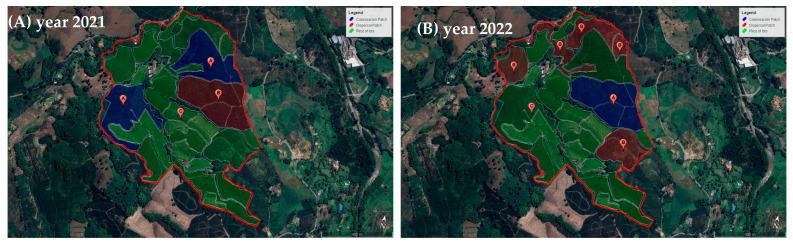
San José farm Release site (**A**). Year 2021 and (**B**). Year 2022. (A-blue): CBB-colonization coffee plots, (B-red): CBB-dispersal coffee plot, (C-green): Plots preparing for the third, fourth, and fifth year of harvest and other crops systems.

**Figure 3:**
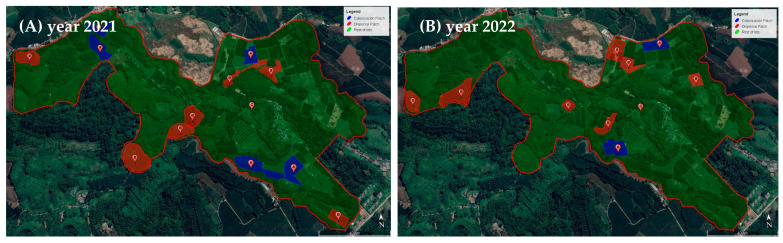
Naranjal Experimental Station No release site. (**A**). Year 2021 and (**B**). Year 2022. (A-blue): CBB-colonization coffee plots, (B-red): CBB-dispersal coffee plot, (C-green): Plots preparing for the third, fourth, and fifth year of harvest and other crops systems.

At each farm, San José Release and Naranjal No Release, coffee plots older than six years of age that had been renovated (via stumping) were identified and labeled as dispersal plots. Coffee plantations less than 24 months old and planted no more than 200 m away from dispersal plots were selected as the most suitable colonization plots for CBBs (Figure 2 and Figure 3). Coffee plots that were sources of dispersing CBB adults (dispersal plots), plus the new areas into which these beetles moved (colonization plots), make up the metapopulation of CBB at the farm level [29], and the area-wide pilot test assessed impact for releasing parasitoids in both types of areas in the San Jose Release farm.

#### 2.1.4. Description of Parasitoid Releases

**Figure A1:**
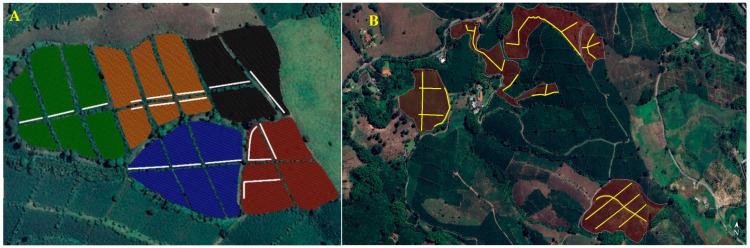
*Prorops nasuta* release routes in the dispersion plots (stumped trees), (**A**): Release routes in the year 2021 (white), (**B**): Release routes in the year 2022 (yellow).

**Figure A2:**
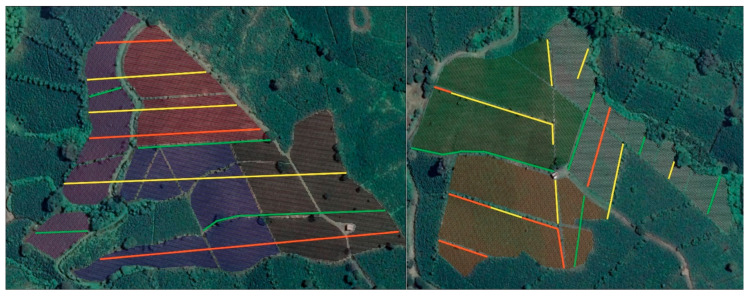
Releases routes for *Phymastichus coffea* and *Prorops nasuta* in the colonization coffee plots year 2021; yellow line (week 1 release); green line (week 2 release); orange line (week 3 release). The routes were repeated every three weeks.

*P. nasuta* was released in the dispersal plots following the paths showed in figure (Appendix A, Figure A1), allowing the wasps to fly from the center and move to their desired direction, expecting coverage and parasitization of CBBs throughout. For the releases of *P. coffea* and *P. nasuta* in the colonization coffee plots, three routes were delimited based on the flight capacity of the wasp (60 and 700 m, respectively) [30,31] and the spatial and topographic characteristics of the plots (Appendix A, Figure A2).

### 2.2. Estimation of Pest Density and Parasitism in the Dispersal Coffee Plots

The CBB density at the dispersal plots and the number of parasitoids used for each release were determined to calculate the host parasitoid ratio.

**Figure 4:**
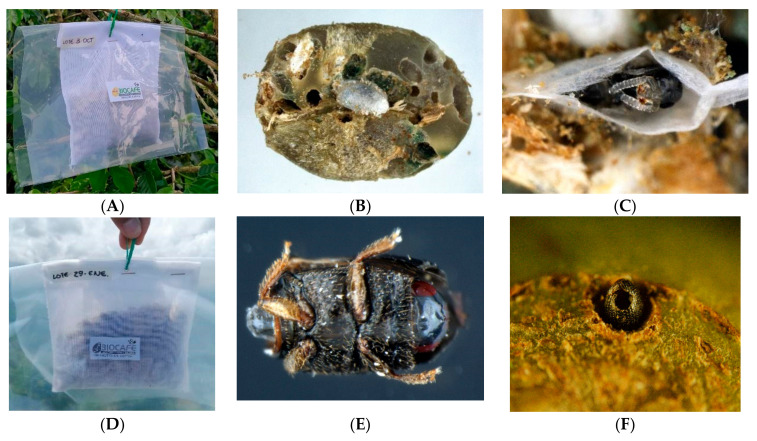
*Prorops nasuta* and *Phymastichus coffea* wasps for release. (**A**): Tulle fabric bag with parchment coffee seeds infested with *P. nasuta-* parasitized CBB, (**B**): Pupal cocoons of *P. nasuta* inside parchment coffee seeds infested with CBB, (**C**): Adult *P. nasuta* inside the pupal cocoon before emerging. (**D**): Tulle fabric bag with *P. coffea*-parasitized CBB on parchment coffee beans, (**E**): Pupa of *P. coffea* inside CBB, (**F**): parasitized CBB adult in a field coffee berry with the emerging hole made by the *P. coffea* adult.

*Prorops nasuta* individuals were released as adults inside parchment coffee seeds infested with parasitized CBBs contained in a tulle fabric bag, two weeks before renovation (stumped trees) in the dispersal coffee plots at the San Jose Release farm (Figure 4A–C). The percentage of parasitism and the number of wasps per seed were estimated based on observation units of 50 seeds randomly selected from the mass-rearing production for each releasing event to estimate the number of adult parasitoids in each release. A total of 1276 seeds were examined. To estimate the number of infested coffee berries in the release plots, 500 coffee plants were selected through random sampling. A field map was created for each plot, and the position of each tree within the plot was identified. The random number selection function in Excel was used to choose the plants. The selected plants were marked with a yellow plastic tape, and each plant’s productive branches were counted. The total number of CBB-infested and -uninfested berries was recorded in the branch containing the most coffee berries. The total number of infested berries per plant and infested berries in the whole dispersal coffee plot was calculated by multiplying this value by the total coffee plants in the release plot. Then, the host:parasitoid ratio was calculated based on the number of infested coffee berries in the plot and the total number of wasps recorded.

### 2.3. Impacts of P. nasuta Releases in the Dispersal Coffee Plots

The impact of *P. nasuta* releases on the density of the CBB population and the dispersal capacity of CBB adults after plant stumping was estimated. Moreover, the initial CBB infestation levels in both farms, the San Jose Release site and the Naranjal No Release site, was taken; for this, randomly selected plants were taken and the most productive branch in the trees’ middle third was chosen to count the total number of fruits and the infested ones. Afterwards, the CBB population in the coffee berries remaining on the ground after the coffee plants were stumped were estimated. To perform this, a 0.5 m^2^ rectangle was placed in 60 randomly selected points, then all the coffee berries on the ground within were collected and taken to the laboratory for dissection. So, the total number of CBB life stages per plot was determined.

### 2.4. Estimation of Pest Density and Parasitism in the Colonization Coffee Plots

The CBB density at the colonization plots and the number of parasitoids used for each release were determined to calculate the host:parasitoid ratio.

Releases of *P. coffea* in the colonization coffee plots (coffee plants of 18 months of age) at the San Jose Release farm started once the berries in the major areas of the farm had a dry matter content greater than or equal to 20%. This percentage is typically reached approximately 120 days after coffee flowering, starting around March each year. *Phymastichus coffea* was released as adults inside parchment coffee seeds infested with parasitized CBBs contained in a tulle fabric bag (Figure 4D–F).

**Figure 5:**
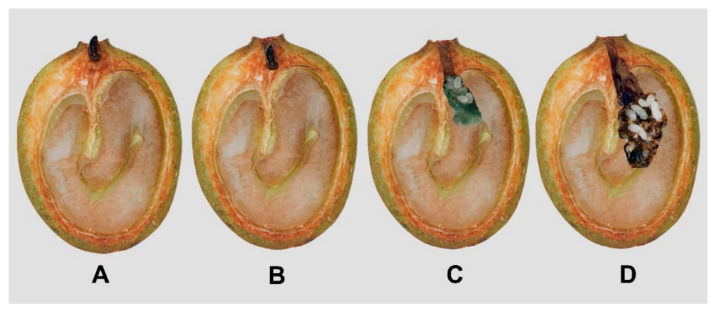
Positions of CBBs boring into coffee fruits, (**A**): CBB is initiating penetration into the fruit, (**B**): CBB is in the penetration tunnel, (**C**): CBB perforates the bean and creates the oviposition chamber, (**D**): CBB inside the coffee bean with offspring [10].

The percentage of parasitism and the number of wasps per seed were obtained to estimate the number of *P. coffea* parasitoids released. For this, 10 seeds randomly selected from the mass rearing production for each releasing moment for a total number of 540 were evaluated. The density of infested coffee berries was also calculated to estimate the ratio of wasps released to infested coffee berries. To perform this, for each plot, in 120 coffee plants monthly selected through random sampling as described in Section 2.2, the number of branches with berries per plant were counted. Therefore, on the most productive branch, the total number of berries was counted, as well as the number of berries showing CBBs in boring positions A + B [10] (Figure 5). From these data, the total number of CBB-infested berries per plot was estimated. The ratio of wasps to infested coffee berries was then calculated based on the total number of parasitized CBBs on parchment coffee and the total infested coffee beans with CBBs in A + B in the whole coffee plot.

### 2.5. Estimation of Parasitism Rates for P. coffea and P. nasuta in the Colonization Coffee Plots

The rate of parasitism (%) caused by *P. coffea* and *P. nasuta* on the CBBs was determined bi-weekly from coffee fruits collected in the colonization coffee plot of the San Jose Release farm. A total of 96 infested coffee berries with entry positions of the borer into the fruit (A + B) (Figure 5) for *P. coffea*, and from 288 in positions inside the fruit (C + D) (Figure 5) for the *P. nasuta*. The numbers of CBB or parasitized CBB life stages were recorded by dissection.

### 2.6. Impact of P. coffea and P. nasuta in the Colonization Coffee Plots

After the monthly release of *P. coffea* (between March and July 2021 and March and June 2022 for a total of 54 releases, 120 coffee plants were randomly selected, using the methodology described in Section 2.2, for two years), the number of productive branches was chosen on each plant, and the most productive branch in the middle third was selected. The total number of fruits and the number of fruits infested by the CBB were then counted on this selected branch. The CBB-infested berries were collected, dissected, and the numbers of CBB life stages per berry were recorded. From this information, the average number of infested berries and the population of CBB per branch per plant were estimated.

### 2.7. Statistical Analysis

In the dispersal coffee plots, the average and standard errors were estimated for the variables: number of total fruits per productive branch, infested fruits per branch, and number of CBB life stages (eggs, larvae, pupae, and adults) within infested coffee berries per m^2^. In the colonization coffee plots, the average and standard errors were estimated for the variables: number of CBB-infested berries per branch, number of life stages of CBB borer per productive branch over time, and CBB infestation per productive branch over time; subsequently, a one-way analysis of variance was performed, the means were compared between years and farms, San José (Release) and Naranjal (No Release), using the least significant difference test at 5%. The analyses were performed using the statistical program SAS (Statistical Analysis System) version 9.4 [32].

## 3. Results

### 3.1. Size of Dispersal and Colonization Coffee Plots in Study Areas

In 2021, in the San Jose Release farm, there were 5.7 ha classified as dispersal plots (from which CBBs would move out) and 8.9 ha of newly colonized areas (first harvesting preparation coffee crops). In 2022, there were 11.4 ha and 5.7 of dispersal and colonizing plots respectively. For comparison purposes, the Naranjal Experimental Station No Release site had 6.4 ha of dispersal areas and 4.1 ha of newly colonized coffee plots in 2021, and in 2022, these values were 5.9 ha of dispersal plot and 5.8 ha of newly colonized coffee plots.

### 3.2. Pest Density and Parasitism in the Dispersal Coffee Plots

In January 2021, 67,500 infested parchment coffee seeds containing CBB life stages parasitized by *P. nasuta* were placed in the coffee trees of the San Jose Release farm’s dispersal plots (5.7 ha). These CBBs parasitized in parchment coffee seeds had a parasitism rate of 72.8% ± 4.8, and from 13 evaluations of 50 seeds each, an average of 447 ± 62 adult parasitoids were expected to fly away per observation unit. Thus, the estimated number of wasps released was calculated to be 567,397 ± 10,091. In January 2022, 80,000 infested parchment coffee seeds containing CBB life stages parasitized by *P. nasuta* were released in the new dispersal plots (11.4 ha). These CBB parasitized in parchment coffee seeds had a parasitism rate of 79.4% ± 6.8, and from 6 evaluations of 50 seeds each, an average of 578 ± 63 adult parasitoids was estimated per observation unit. The estimated number of wasps released was 924,444 ± 23,236. The average number of infested berries per coffee tree in the San Jose Release dispersal plot was calculated to be 27.3 ± 2.3 in 2021 and 4.0 ± 0.1 in 2022. Furthermore, for a total 34,974 coffee trees the first year and 50,050 trees during the second one, the total estimation was 954,790 and 200,200 infested berries in the entire dispersal plot in 2021 and 2022, respectively. Thus, the inferred release ratio for *P. nasuta* was estimated as one wasp per 1.7 infested berries in 2021 and one wasp per 0.2 in 2022.

### 3.3. Impact of P. nasuta Releases in the Dispersal Coffee Plots

The impact of *P. nasuta* releases in the dispersal plots was evaluated based on their ability to reduce CBB populations before they dispersed to other coffee plots. The data from the evaluation prior to plant stumping showed that in both farms the average numbers of total fruits per productive branch and infested fruits per branch were statistically similar (Table 1). This means that the dispersal plots in both farms had the same potentially dispersing CBB population at the beginning of the experiment.

After the releases of *P. nasuta* in 2021, two months later, the average number of CBB stages within coffee berries on the ground per m^2^ was calculated to be 57.6 ± 13.3 at the San Jose Release farm compared to 303.6 ± 45.1 at the Naranjal No Release Experimental Station (Table 2). This is an 81% decrease in the CBB population as a mean of referring to lowering the dispersal rate at the San Jose Release farm compared to the Naranjal No Release Experimental Station, where *P. nasuta* was not used. Ground populations of CBB within infested berries in 2022 showed no differences between farms (Table 2).

### 3.4. Pest Density and Parasitism in the Colonization Coffee Plots

Between March and July 2021, 116,600 parchment coffee seeds containing CBB adults parasitized by *P. coffea* were released at the San Jose Release farm’s colonization coffee plots (8.9 ha). These CBB adults had a parasitism rate of 67.7% ± 5.1, and from 29 evaluations of 10 seeds each, an average of 35 ± 4 adult parasitoids were counted. The estimated number of *P. coffea* wasps released on these 8.9 ha was 407,200 ± 85,270 (Table 3).

In 2022, between March and July, 142,000 parchment coffee seeds containing CBB adults parasitized by *P. coffea* were released at the San Jose Release farm’s colonization coffee plots (8.9 ha). These CBB adults had a parasitism rate of 66.5% ± 6.2, and from 25 evaluations of 10 seeds each, an average 28 ± 4 adult parasitoids was counted. The estimated number of *P. coffea* wasps released on these 8.9 ha was 408,690 ± 103 (Table 3).

Between March and June 2021, the maximum average of infested berries per tree with CBBs penetrating the fruit (positions A + B) (Figure 5) was 20 ± 4.6, with an estimated total of 1,278,600 infested berries in a total 63,930 coffee trees. For 2022, the average number of infested berries was 37.4 ± 2,3, with an estimated total of 2,390,982. With this information, we calculated that the ratio of female *P. coffea* released per number of infested coffee berries in the colonization coffee plots was 1:3 in 2021 and 1:6 in 2022.

In June 2021, 71,017 parchment coffee seeds containing CBB life stages parasitized by *P. nasuta* were released at the San Jose Release farm’s colonization coffee plots (8.9 ha). These CBBs parasitized in parchment coffee seeds had a parasitism rate of 58%, and from 10 evaluations of 50 seeds each, an average of 213 ± 39 adult parasitoids were estimated per observation unit (50 seeds). The estimated number of *P. nasuta* wasps released on these 8.9 ha in the colonization coffee plots was 302,259 ± 9346.

In June 2022, 50,000 parchment coffee seeds containing CBB life stages parasitized by *P. nasuta* were released at the San Jose Release farm’s colonization coffee plots (8.9 ha). These CBBs had a parasitism rate of 84.9% ± 6.0, and from 5 evaluations of 50 seeds each, an average of 461 ± 122 adult parasitoids were estimated per observation unit. The estimated number of *P. nasuta* wasps released on these 8.9 ha in the colonization coffee plots was 461,313 ± 21,449.

In June 2021, the maximum average of infested berries per tree with CBBs inside the bean (positions C + D) (Figure 5) was 14.5 ± 0.1, with an estimated total of 829,023 berries infested by CBBs in the colonization coffee plots in 57,174 coffee trees. In 2022 it was 17.8 ± 0.1 and 326,060 berries infested. The ratio of *P. nasuta* females released per number of infested coffee berries in the colonization coffee plots was 1:2.7 in June of 2021 and 1:0.7 in June of 2022.

### 3.5. Parasitism Rates for P. coffea and P. nasuta in the Colonization Coffee Plots

**Figure 6:**
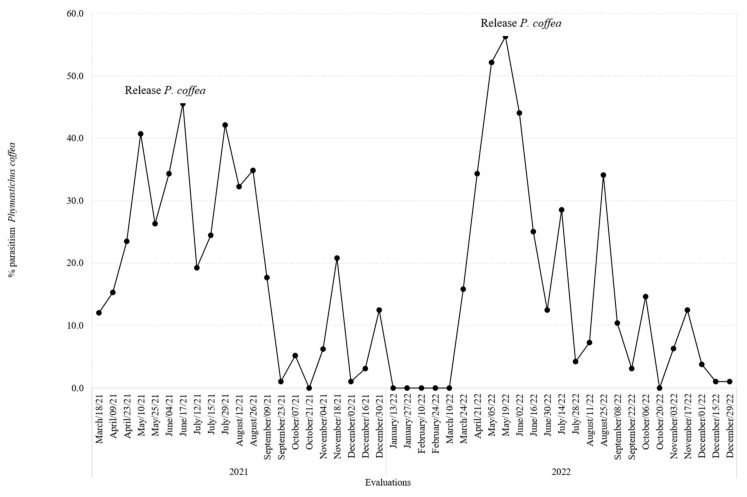
Percentage parasitism over time of *Phymastichus coffea* in the colonization coffee plots of the San Jose Release farm.

**Figure 7:**
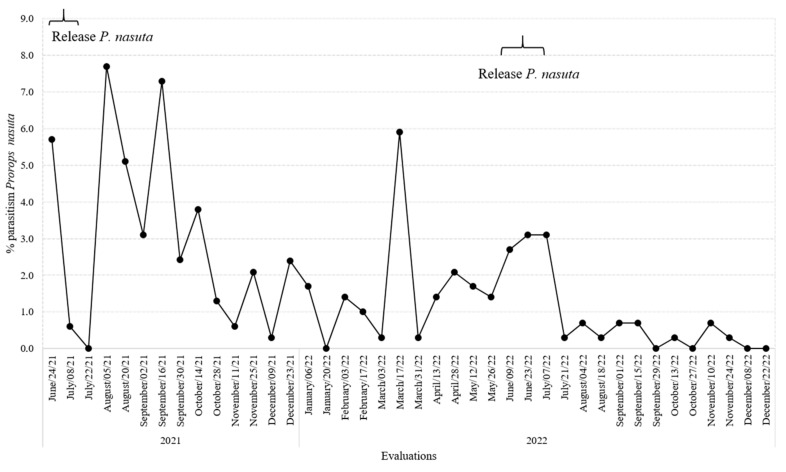
Percentage parasitism over time by *Prorops nasuta* in the colonization coffee plots of the San Jose Release farm.

The percentage of parasitism in the field by *P. coffea* over time peaked at 56.3% in 2022, the second year of this evaluation (Figure 6). However, the inability of this species to establish permanent field populations in Colombia was confirmed, as its level of parasitism decreased to zero within five months of its last release (Figure 6). For *P. nasuta*, field parasitism rates were lower, reaching a peak of 7.7% (Figure 7).

### 3.6. Impact of P. coffea and P. nasuta in the Colonization Coffee Plots

On the San Jose Release farm, the wasps released in 2021 reduced the number of CBB-infested fruits per branch by 77.5%. For the year 2022, the reduction was by 51.1% compared to the Naranjal No Release Experimental Station, where no wasps were released (Table 4).

The impact of *P. coffea* releases started to be evaluated in the first forming harvest coffee crops, at the time, the CBB population in January and February 2021 was zero (Table 5), as there were no suitable fruits for CBB infestation yet. In March, the Naranjal No Release Experimental Station started with higher populations of CBBs than the San Jose Release farm, as a possible consequence of the lower number of CBBs dispersing from the stumped coffee trees demonstrated by the effect of *P. nasuta* releases in reducing the populations from the dispersal plots. In other words, the releases of *P. nasuta* helped decrease the CBB populations that were going to disperse. The difference between the San Jose Release farm and Naranjal No Release Experimental Station can be observed between March and August, which corresponds to the period when the harvest fruits were forming. From September onwards, the harvest was carried out, removing also both the CBBs and the wasps, making difficult to directly evaluate the wasps’ impact.

Statistical differences were observed between the San Jose Release farm and Naranjal No Release Experimental Station from March to August 2021 in the number of CBB life stages per branch per tree (Table 5) due to the impact of *P. coffea* and *P. nasuta* releases. After that, other dynamics came into play regarding CBB population within a coffee plantation, as harvesting takes place and new fruits from other flowering events begin to form the following year’s intermediate harvesting and the same year’s main harvest. However, *P. coffea* releases continued in this plot during the second year.

In eight out of the twelve months of 2022, there were differences in populations between the San Jose Release farm and Naranjal No Release Experimental Station, with lower CBB numbers in San Jose (Table 5). The four months with no statistical differences correspond to April and May when the intermediate coffee harvest was collected, and during August and September when the main coffee harvest was collected. During the rest of the time, there were fewer CBBs on the San Jose Release farm compared to the Naranjal No Release Experimental Station as a result of the releases of the two parasitoid species.

**Figure 8:**
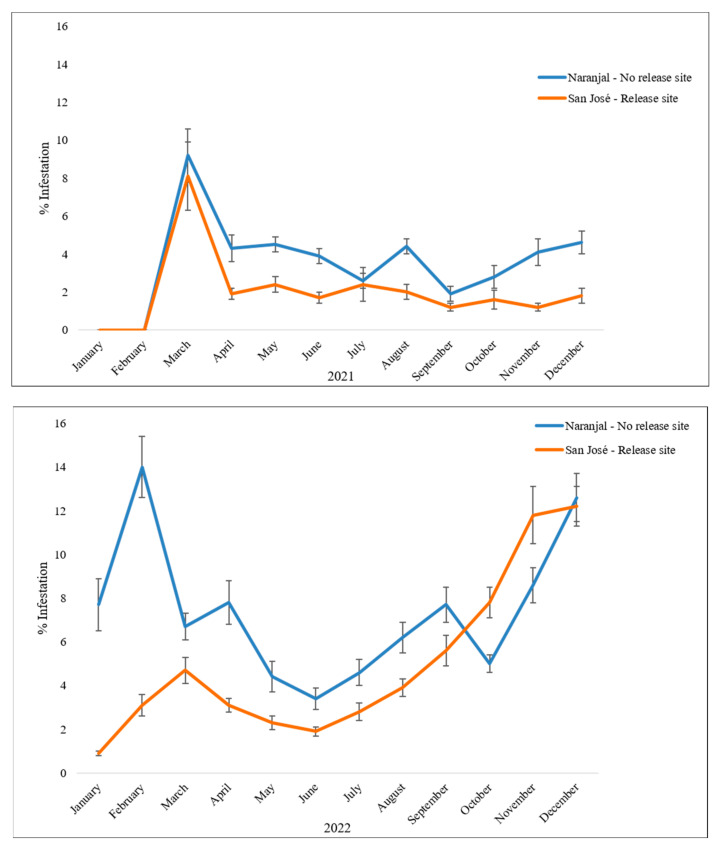
CBB infestation (mean ± SE) per productive branch over time in the Colonization coffee plots of each evaluated farm in 2021 and 2022. Bars indicate Standard Error.

The percentage of CBB infestation per productive branch was lower on the San Jose Release farm. In 2021, both farms started without infestation in January and February because the fruits were not yet suitable for CBB attack. From March onwards, the infestation percentages on the San Jose Release farm were lower compared to those at the Naranjal No Release Experimental Station due possibly to the parasitoid releases, except for the month of July, September, and October, when the percentage of infestation was similar. In 2022, from January to September, there were differences in the infestation percentage between the San Jose Release farm and Naranjal No Release Experimental Station, with lower levels in San Jose (Figure 8). In October, November, and December, the infestation levels increased in both farms due to the natural dynamics of the insect.

## 4. Discussion

The area-wide IPM system examined in this pilot test is based on recognition of the metapopulation structure of CBB populations, with some coffee areas being sources of CBB dispersal and others serving as their colonization areas (i.e., source and sink). This population structure for the CBB occurs in coffee plantations in Colombia because 20% of a coffee plantation is renovated annually. The part of the farm where coffee trees are removed and replanted provides a source of beetles that move out of the renovated coffee plot and colonize other plots, especially those preparing berries for the first year of coffee harvest [10]. Several factors in the biology of the CBB lead to this population structure in the field. First, the renovation of coffee trees is the agronomic practice that causes the largest number of beetles to disperse because it forces the insects to move from areas in which populations have had at least five years of continuous cycles of generations’ to build up in size [12,33]. Also, the beetles displaced from such renovated plots can survive more than 155 days, and the majority of the adults flight during the first 70 days [12]. These conditions allow displaced CBB adults sufficient time to reach adjacent coffee plantations when they are in their first coffee harvest. Studies have shown that CBB adults can sustain continuous flight for up to 90 min, and migrating adults can become entrapped and moved by air currents over distances of at least 500 m during that time [13].

In this study, the evaluation was carried out in first-harvest coffee plots because they were producing coffee berries for the first time and had no CBB population before. However, within a coffee farm, CBB colonization would also occur in second, third, and fourth harvest-year plots, especially those with the most mature coffee berries [34,35]. Using only first-harvest plots allowed us to demonstrate the effects of management (parasitoid releases) on colonization rates without the need to use marked insects. As a practical control strategy, the release of parasitoids would need to be made in first-, but also second- and, if possible, third-year harvest plots, depending on the production cost of the parasitoids.

The area-wide IPM strategy employed in this study was effective for CBB management in the coffee production systems in Colombia. If applied in other regions, the production systems would need to be analyzed to determine if they also recognize dispersal and colonization patches to identify opportune moments to release CBB parasitoids, then reduce CBB populations. In our study, releases of *P. nasuta* in the dispersal plots reduced the numbers of CBBs by up to 81%, and, therefore, releases should have reduced the dispersal rate. Studies in Colombia by Bacca [22] demonstrated that *P. nasuta* immediately reduces CBB populations, as it can kill all CBB stages by acting as either a predator or parasitoid, even when the parasitism level is low; when the female of *P. nasuta* penetrates the borer-infested berry, it paralyzes the CBB female caring for the CBB beetle’s offspring, then feeds on the CBB eggs and first-instar larvae and deposits its eggs on the CBB second-instar larvae, prepupae, and, to a lesser extent, on pupae. After releases, *P. nasuta* adults remain in the field plots for weeks since adult females have an average longevity of 28 days, during which they feed on the immature stages of the CBB [36]. Studies in Colombia by Bacca [22] demonstrated that *P. nasuta* immediately reduces CBB populations, as it can kill all CBB stages by acting as either a predator or parasitoid. The results of Bacca’s [22] field studies suggest that *P. nasuta* is the most promising agent for biological control of the CBB in Colombia. In the present study, the contributions of parasitism and predation to overall mortality by *P. nasuta* were not separated. This parasitoid efficiently reduced the CBB population inside berries before coffee plantation renovation. Ideally, for optimal CBB control using *P. nasuta*, releases should be made at least one or two months before the coffee plot renewal, because while *P. nasuta* has demonstrated a good ability to search for the CBB in coffee berries of live plants and persist, it does not do so once the coffee berries have matured and fallen to the ground. Previous studies showed that adults of *P. nasuta* can disperse up to 700 m in search of CBB life stages to parasitize [31]. We believe that the effectiveness of *P. nasuta* in reducing CBB populations in dispersal plots is due to its high specificity for the CBB, its predatory behavior, and its longevity. If this species cannot be used in other regions, it is suggested to evaluate generalist predators such as *Cathartus quadricollis* (Guérin-Méneville, 1829) and *Ahasverus advena* Waltl, 1834 (Coleoptera: Silvanidae), which reduced CBB populations under laboratory conditions by up to 69% and 63%, respectively [20]. Liang and collaborators [37] have also reported predation of CBBs by *C. quadricollis* ranging 12 to 49% in Hawaii, and recently, Follet et al. [38] developed a breeding station for increasing this species in a field conservation biology approach to control CBB in Hawaii.

In our study, the second parasitoid tested, *P. coffea*, was very efficient in reducing the colonization rate in coffee plots at the time of first or second harvest. The success of *P. coffea* is likely due to its high specificity to the CBB. In mass rearing of *P. coffea* for releases, a host:parasitoid ratio of 1:10 is recommended [27]. However, Benavides et al. [16] suggested the use of one wasp for every five coffee berries infested by CBBs during the stages when they enter the fruit (A + B) (Figure 5). In our study, a ratio of one female wasp per three to four infested berries was used as the release rate. If a 1:5 parasitoid:infested berry standard were adopted, either a larger area could have been treated or a smaller number of wasps could have been used. This choice of parasitoid:infested berry ratio for releases will depend on the parasitoid species used and the cost of mass rearing it.

Our study lasted two years, and the results suggest that mass releases have promise for control of the CBB over large areas. However, this area-wide IPM strategy should work better if it were implemented gradually until the entire area under study was covered as follows: in year 1, 20% of the dispersal plots and 20% of the colonization coffee plots (which includes the areas of the first coffee harvest), should be treated with parasitoid releases, for a total of 40% of the whole farm. In year 2, the dispersal plot from the first year would become the colonization coffee plot for the second year and would be targeted with *P. coffea* releases, so that 20% of the new dispersal plots would be treated with *P. nasuta* releases. This latter strategy is applied during the third and fourth years to cover the entire farm area in coffee. In this way, both a reduction in colonization of new plots and in the level of dispersal of the CBB could be achieved using the two parasitoids in concert. However, more conclusive results will be obtained from the fourth year onwards. Monitoring the population dynamics of the CBB and its parasitoids would allow for the strategy to be modified as needed. For the second coffee harvest and beyond, all management strategies of the CBB should be integrated, including the optional application of insecticides and the continuous use of a natural enemy, the fungus *Beauveria bassiana* (Bals.-Criv.) Vuill., at opportune moments, i.e., when the CBB-infestation action threshold is above 2% and monitoring has shown that half of all CBB adults are flying [10]. In addition, cultural control measures, such as timely coffee harvesting and re-picking after each harvest (strip picking if only one main harvest occur), should be employed consistently [10]. Due to the fact that in first and second harvest coffee plantations, there are usually not enough CBBs, chemical control is not traditionally employed; however, if parasitoid releases are needed to be carried out in young coffee trees, a period of at least 30 days should be taken into consideration after and before application [39].

La Niña and El Niño climatic events affects CBB population size as the first brings higher temperature and less rain on Colombian coffee areas. Furthermore, El Niño relates to higher CBB infestation levels, contrary to La Niña. The results of our study showed that releases of *P. nasuta* were very successful in reducing the dispersal of CBBs during the first year, despite being at the beginning of a La Niña climatic event and coming from the transition of a neutral climatic event. During the second year, in which the La Niña event continued, the populations of the CBB in the dispersal plots were not as high, so the control effect of *P. nasuta* was not seen. We attribute this outcome to the rainy periods in this time, which caused infested coffee berries that had fallen to the ground to decompose more quickly, killing more CBB life stages due to the resulting lack of food. Consequently, the development and emergence of the CBB was lower. Based on these results, It is recommended not to release parasitoids under these conditions and to re-allocate these wasps to later releases during the main harvest. This conclusion implies that parasitoids would not be released during crop renovating periods but, rather, parasitoids would be released when coffee berries are forming, as was the case for the second release of this species in this study. Despite variation in weather, *P. coffea* successfully reduced CBB infestation rate, which ranged from 51 to 77% (Table 4). Based on the above, it is expected that during a Neutral or El Niño year, both the parasitism rates and the number of generations of *P. coffea* would be higher due to higher temperatures and shorter generation times [40]. We expect that the parasitoid *P. coffea* will perform better under conditions of higher temperatures and less rainfall, as occurs during an El Niño event. However, this should be evaluated further under laboratory and field conditions [40].

In Colombia, we propose that the area-wide IPM strategy be used in areas with high vulnerability to CBB infestation and crop loss. For the specific case of the Department of Caldas, in a neutral year, 3.9% of the coffee-growing area has a high vulnerability to the CBB, while in an El Niño event, it can increase to 15.7% [41]. During a La Niña event, only 2.6% of the planted area is at high risk. Identification of the high-risk areas allows the production of CBB parasitoids to be used in a rational manner to reduce populations of CBBs where needed most. It should be noted that *P. nasuta* is an aggressive wasp with a high predation capacity, and has been shown to establish well under field conditions in Colombia, even with low parasitism levels reported. A single release is sufficient to establish the wasp in the field [23]. This means that if it is released every year in renovating coffee crops, it will decrease the population on the designated area and keep working permanently in surrounding coffee crops, reducing dispersal processes in the dispersal plots and in the productive plots.

*Phymastichus coffea* is a highly specific, efficient parasitoid of CBB adults that attacks CBB beetles as they colonize new coffee berries. The parasitization process only lasts between two and three days because adult females have a short lifespan [30]. However, *P. coffea* does not establish permanent populations in the field in Colombia. In this study, it was demonstrated that after five months without releases, this parasitoid could no longer be found in the field. This means that releases of *P. coffea* can be expected to reduce CBB populations during the pest’s colonization of the berries, but releases would need to be made repeatedly. For *P. coffea* to establish itself in the coffee landscape, it may need alternate hosts.

Finally, given that the objective of the area wide IPM program against the CBB in Colombia is CBB suppression over the medium- to long-term, the release of parasitoids would have to be performed for periods of five years or more. To do so, the availability of wasps and their production cost should be taken into consideration. As a single wasp can be acquired for a cost of USD 0.01 (Biocafé, this research exclusive contract), from the perspective of the ideal ratio of one *P. coffeea* per ten CBB-infested coffee berry with the insect in boring position (A + B), and considering the cost per pound of coffee in USD 1.24 which contains around 2300 coffee seeds, releasing wasps to save coffee beans would not appear to be economically feasible. However, if decreasing CBB colonization rates in the field would cut its population dynamics during berry growing by means of avoiding two to three exponential generations of CBBs, then the cost of the parasitoids would impact the economics of the coffee production at farm level since harvest quality is secured. This statement is aligned with the economical analysis by Duque and Baker [11], indicating that the economic losses, calculated as the income not received by the coffee farmers due to quality reduction for the infestation levels on the coffee parchment, would be, at a national level in Colombia, USD 15 million if infestation level is 3%, but USD 66 million if the level goes up to 10%. Even though the cost of releasing parasitoids would positively impact the economics of the growers, attempts to reduce the cost of rearing the wasps are recommended.

## 5. Conclusions

The release of *Prorop nasuta* decreased the dispersal of the CBB from stumped coffee plantations and *Phymastichus coffea* complemented to slow down the colonization process in the young coffee planted nearby. In this way, the recommendation resulting from this research is: to implement an area-wide IPM biological control strategy using African parasitoids to minimize the dispersal process of CBBs from stumping old coffee plots, and to complement decreasing the rate of CBB colonization in younger coffee plantations nearby; together, both methods will reduce pest pressure. But older coffee crops preparing the third, fourth, and fifth harvests, will require a complete integrated pest management program for CBB, which is based on cultural control by means of timely harvest and re-picking twice a year once harvesting seasons end, and is complemented with the use of biocides such as the fungus *B. bassiana* or with chemical insecticides at the appropriate times. 

## Figures and Tables

**Table 1:** Mean numbers of total fruits per productive branch and infested fruits per branch in the dispersal plots (stumping trees) at the San Jose (Release) farm and Naranjal (No Release) Experimental Station prior to plant removal (stumping).

Farm	Num. Trees	Berries/Branch	CBB-Infested Berries/Branch
		Mean (±SE)	Mean (±SE)
Naranjal-No Release Site	300	28.7 ± (1.3) A	8.5 ± (0.5) A
San Jose-Release Site	200	29.3 ± (1.4) A	6.4 ± (0.4) A

For each column, means followed by the same letter were not significantly different by LSD at alpha = 0.05.

**Table 2:** Average number of CBB life stages (eggs, larvae, pupae, and adults) within infested coffee berries per m^2^ collected from the ground in the dispersal plots at the San Jose (Release) farm and the Naranjal (No Release) Experimental Station.

Year	Farm	Number (Mean ± SE) of CBB Life Stages Per m^2^
		Mean ^&^
2021	Naranjal—No Release Site	303.6 ± (45.1) A
	San Jose—Release Site	57.6 ± (13.3) B
2022	Naranjal—No Release Site	8.7 ± (2.7) A
	San Jose—Release Site	10.2 ± (2.0) A

Eggs, larvae, pupae, and adults. ^&^ For each year, means followed by different letters were significantly different by LSD test at alpha = 0.05 (Fc = 19. 08, *p* ≤ 0.0001).

**Table 3:** Number of seeds with *Phymastichus coffea*-parasitized CBB and number of parasitized CBBs released in the colonization coffee plots.

Year	Month	Coffee Seeds with CBB Parasitized	Released Wasps
2021	February	35,400	123,600
	March	37,200	130,000
	April	16,000	55,900
	May	12,000	41,900
	June	12,000	41,900
	July	4000	13,900
2022	March	45,500	127,400
	April	34,500	96,600
	May	18,500	48,100
	June	37,000	116,180
	July	6500	20,410

**Table 4:** Mean (±SE) number of CBB-infested berries per branch in the Colonization coffee plots.

Year	Farm	Number of CBB-Infested Berries Per Branch
		Mean ± (SE)
2021	Naranjal-No Release Site	78.6 ± (11.2) A
	San Jose-Release Site	17.7 ± (3.2) B
2022	Naranjal-No Release Site	90.4 ± (11.2) A
	San Jose-Release Site	44.2 ± (6.9) B

For each year, means followed by different letters were significantly different by LSD test at alpha = 0.05. (year 2021 Fc = 22.21, *p* ≤ 0.0001; year 2022 Fc = 13.08, *p* ≤ 0.0007).

**Table 5:** Mean (±SE) number of life stages of CBB per productive branch over time in the Colonization coffee plots.

		Farm				
Year	Naranjal-No Release Site			San Jose-Release Site		
	Month	Mean CBB Life Stages/Branch (±SE)		Month	Mean CBB Life Stages/Branch (±SE)	
2021	January	0.0 ± (0.0) A		January	0.0 ± (0.0) A	
	February	0.0 ± (0.0) A		February	0.0 ± (0.0) A	
	March	11.7 ± (2.4) A		March	0.9 ± (0.2) B	
	April	7.9 ± (1.9) A		April	1.2 ± (0.3) B	
	May	11.7 ± (1.5) A		May	4.8 ± (1.1) B	
	June	11.9 ± (1.7) A		June	3.7 ± (1.0) B	
	July	13.4 ± (1.9) A		July	6.4 ± (1.7) B	
	August	29.0 ± (3.3) A		August	7.8 ± (1.7) B	
	September	6.7 ± (1.1) A		September	4.1 ± (0.9) A	
	October	5.9 ± (1.3) A		October	2.3 ± (0.6) B	
	November	5.9 ± (1.1) A		November	1.3 ± (0.4) B	
	December	3.4 ± (0.7) A		December	1.9 ± (0.5) A	
2022	January	4.7 ± (0.8) A		January	0.8 ± (0.2) B	
	February	17.2 ± (1.9) A		February	3.3 ± (0.6) B	
	March	11.6 ± (1.2) A		March	2.1 ± (0.4) B	
	April	3.7 ± (0.7) A		April	5.5 ± (1.0) A	
	May	5.3 ± (1.0) A		May	7.4 ± (1.3) A	
	June	6.3 ± (1.1) A		June	2.3 ± (0.5) B	
	July	11.5 ± (1.8) A		July	7.2 ± (1.2) B	
	August	7.7 ± (1.1) A		August	5.8 ± (0.9) A	
	September	18.7 ± (2.2) A		September	15.2 ± (2.0) A	
	October	16.8 ± (2.3) A		October	9.8 ± (1.3) B	
	November	11.0 ± (1.6) A		November	5.4 ± (0.9) B	
	December	14.9 ± (1.9) A		December	11.8 ± (2.0) B	

Different letters imply significant differences between farms every month according to the least significant difference test at 5%. Letters are compared between columns. (Naranjal year 2021 Fc = 15.47, *p* ≤ 0.0001; year 2022 Fc = 11.31, *p* ≤ 0.0001; San José year 2021 Fc = 6.63, *p* ≤ 0.0001, year 2022 Fc = 13.53, *p* ≤ 0.0001).

## Data Availability

The data presented in this study are available on request from the corresponding author.
